# The value of routine histopathological examination after haemorrhoidectomy in patients at low and high risk of anal squamous intraepithelial lesions and cancer

**DOI:** 10.1111/codi.70056

**Published:** 2025-03-17

**Authors:** Panuwat Pornkul, Guy Lampe, Renae Bertucci, Shehan Wickramasinghe, Kerenaftali Klein, Chrispen Mushaya, Shinichiro Sakata

**Affiliations:** ^1^ Division of Colorectal Surgery, Department of Surgery Townsville University Hospital Townsville Queensland Australia; ^2^ College of Medicine and Dentistry James Cook University Townsville Queensland Australia; ^3^ Central Pathology Laboratory, Department of Anatomical Pathology Royal Brisbane and Womens Hospital Herston Queensland Australia; ^4^ Returned and Services League of Australia Brisbane Queensland Australia

**Keywords:** anal cancer, anal dysplasia, anal intraepithelial neoplasia, anal squamous cell carcinoma, anal squamous intraepithelial lesions, haemorrhoidectomy, high‐grade squamous intraepithelial lesions, histopathology, low‐grade squamous intraepithelial lesions, routine histopathology

## Abstract

**Aim:**

Routine histopathological evaluation of haemorrhoidectomy specimens is not ubiquitous amongst surgeons as its utility is debatable. This is the first study to assess the detection rate of anal squamous intraepithelial lesions (aSIL) and anal squamous cell carcinoma (aSCC) in low‐ and high‐risk patients.

**Method:**

This 9‐year retrospective study assessed electronic medical records of all patients who underwent excisional haemorrhoidectomy within an Australian tertiary referral hospital. Patients with sinister clinical examination findings were excluded from the study. Data collected included patient demographics, pertinent history, relevant risk factors, histopathology reports and digital rectal examination findings. Cost–benefit analysis of routine pathology submission and a city‐wide survey of surgeons to ascertain current practices were also undertaken.

**Results:**

The overall prevalence of incidental aSIL and aSCC was 27 (8.1%); 19 patients (5.7%) had low‐grade squamous intraepithelial lesions (LSILs), seven (2.1%) had high‐grade squamous intraepithelial lesions (HSILs) and one patient (0.3%) had aSCC. More than three out of four were detected in low‐risk patients, with most cases being LSIL. Comparing low‐risk and high‐risk patients, the observed incidental detection rate of aSIL and aSCC was 6.8% (95% CI 4.49–10.17) and 23.1% (95% CI 11.03–52.05), respectively. Multivariate logistic regression showed a large, significant association between high‐risk risk factors and detecting aSIL and aSCC (OR 3.76, 95% CI 1.32–10.68, *P* = 0.013). A city‐wide survey of surgeons demonstrated that 28.6% do not request routine histopathological evaluation and 64.3% thought that the prevalence of sinister incidental pathology in haemorrhoids was 1% or less. The total cost of conducting routine histopathological evaluation per patient was $96.80 AUD ($59.20 EUR, $65.30 USD).

**Conclusion:**

Given the non‐negligible incidental detection rate of aSIL and aSCC in both low‐risk and high‐risk patients, coupled with the cost‐effectiveness of histopathological examination, this study suggests that routine histopathological examination should not be restricted solely to high‐risk patients. Further study of the benefit of surveillance following clinical detection in low‐ and high‐risk patients is needed.

**What does this paper add to the literature?**

There is no consensus on routine histopathological evaluation of haemorrhoidectomy specimens. This study demonstrates that the detection rate of anal squamous intraepithelial lesions and anal squamous cell carcinoma is not negligible in low‐ or high‐risk patients. These findings highlight the need for further research to determine the clinical significance of these lesions to guide surveillance strategies.

## INTRODUCTION

The worldwide incidence of anal cancer is rising. In Australia, a 50% increase was reported between 1982 and 2005 [1]. Anal squamous intraepithelial lesions (aSIL), formerly known as anal intraepithelial neoplasia, are dysplastic precursors of anal squamous cell carcinoma (aSCC). Many institutions offer surveillance for aSIL [2], and healthcare resources are typically directed toward high‐risk patients in whom the prevalence of anal dysplasia and the risk of malignant transformation are thought to be greatest [3, 4, 5]. Widely accepted risk factors include previous human papilloma virus (HPV) infection, men who have sex with men (MSM) status, human immunodeficiency virus (HIV) infection, vaginal and cervical intraepithelial neoplasia (VIN and CIN) and immunosuppression [6].

Routine histopathological examination of haemorrhoids may opportunistically detect aSIL and aSCC. However, the detection rate and cost‐effectiveness of opportunistic screening are uncertain and, understandably, this practice is not ubiquitous among surgeons. Most affected patients are thought to be low risk with low‐grade aSIL, but there is no study in the literature which has examined the benefit of formal surveillance programmes for this cohort. This dilemma is now particularly relevant given the growing emphasis on value‐based healthcare and the sensible allocation of limited resources.

The primary aim of this study was to determine the incidental detection rate of aSIL and aSCC in low‐ and high‐risk patients who underwent excisional haemorrhoidectomy. Our secondary aims were to undertake a cost analysis of routine pathology submission and perform a city‐wide survey of surgeons to ascertain their current local practices and their estimated detection rate of incidental sinister pathology in haemorrhoid specimens.

## METHOD

### Participants

This was a single‐institution 9‐year retrospective observational study including all patients above 18 years of age who underwent excisional haemorrhoidectomy between January 2015 and December 2024 at Townsville University Hospital. This is North Queensland's only tertiary hospital and has a referral catchment of 700 000 people.

**FIGURE 1:**
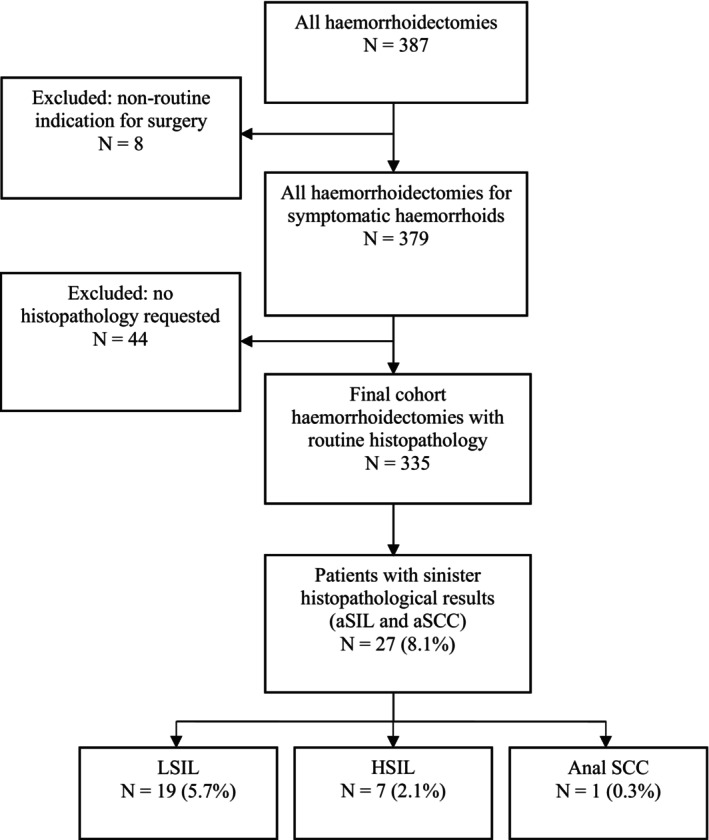
Flow chart of the inclusion and exclusion process and the proportion of patients with incidental histopathological findings. LSIL, low‐grade squamous intraepithelial lesion; HSIL, high‐grade squamous intraepithelial lesion; aSIL, anal squamous intraepithelial lesion; aSCC, anal squamous cell carcinoma.

Patients in this study were identified through relevant procedure codes documented within electronic operating theatre logs. Patients were excluded if their operation was conducted for non‐routine indications, such as for investigating suspicious anal lesions or masses. Patients were also excluded if histopathological analysis was not requested for excised haemorrhoidal tissue (refer to Figure 1).

### Study procedures

Investigators reviewed all electronic medical records to verify operation records, pathology reports, digital rectal examination findings (preoperatively and intraoperatively) and pertinent clinical history. Relevant risk factors such as previous HPV infection, MSM status, HIV infection, VIN/CIN and immunosuppression were determined by medical records and pathology reports. The highest histological grade of dysplasia assigned to each patient was considered the final diagnosis, as this dictated the course of management at our institution. Following the retrospective review of the initial histopathology reports, all cases with abnormal findings were referred for secondary review by an independent board‐certified pathologist, experienced in assessing colorectal pathology. The purpose of this review was to identify reporting discrepancies and to assess diagnostic accuracy within our institution. The results of this secondary review are presented in Table S1 and identify five instances of discrepancies with the initial reported histopathology results, representing a diagnostic revision rate of 18.5%.

Secondary outcomes were assessed by surveying all practising general and colorectal surgeons in Townsville, the largest city in regional Queensland, Australia. Surgeons were asked to state whether they would normally request routine histopathological evaluation of excised haemorrhoidal tissue, in the absence of any suspicious findings on digital rectal examination. Surgeons were then asked to explain the rationale for their practice and to estimate the incidental detection rate of aSIL and aSCC in these patients.

### Statistical analysis

Analysis was completed with R version 4.4.1 for Windows. Results are expressed as median (interquartile range). Qualitative variables are expressed as *n* (%). The Shapiro algorithm was used to determine whether each variable had a normal distribution. The independent Student *t* test or Wilcoxon rank‐sum test as non‐parametric alternatives were considered to compare the statistic of continuous variables in two independent groups. Comparison of frequencies was done by Fisher's exact test due to sample size. A multivariable logistic regression was used to estimate the low‐risk and high‐risk group aSIL and aSCC incidental detection rates, adjusted by age at admission, gender and smoking status. The OR along with the 95% CI was reported. A two‐sided *P* value of less than 0.05 was considered statistically significant.

## RESULTS

From January 2015 to December 2024, 379 patients underwent excisional haemorrhoidectomy for symptomatic haemorrhoids. Of these patients, 335 patients (88.4%) had routine histopathological examination of the excised haemorrhoids. The most common indication for surgery was rectal bleeding (57.3%), and the most common procedure was open excisional haemorrhoidectomy with monopolar diathermy or the LigaSure™ energy device (87.8%). Tables 1 and 2 provide a breakdown of surgical indications and types of haemorrhoidectomy procedures.

**TABLE 1:** Haemorrhoidectomy indication.

Indication	Number (*N* = 335)
Per rectal bleeding	192 (57.3%)
Pain/discomfort	60 (17.9%)
Lump/prolapse	60 (17.9%)
Thrombosis	12 (3.6%)
Other	11 (3.3%)

*Note*: The percentage of the entire cohort is presented in parentheses.

**TABLE 2:** Type of haemorrhoidectomy.

Indication	Number (*N* = 335)
Open haemorrhoidectomy /Ligasure™	297 (87.8%)
Stapled haemorrhoidectomy	2 (0.6%)
Excision and banding	27 (8.1%)
Other, e.g. HAL—RAR and open haemorrhoidectomy	12 (3.6%)

*Note*: The percentage of the entire cohort is presented in parentheses.

Abbreviation: HAL—RAR, haemorrhoidal artery ligation—recto anal repair.

The characteristics of the study cohort are summarized in Table 3. Most of the cohort were considered low‐risk patients by convention; 92.2% did not have clinical history significant for immunosuppression, HIV infection, were not MSM, did not have a known HPV‐associated infection, or prior or ongoing treatment for CIN or VIN.

**TABLE 3:** Characteristics of patients who underwent haemorrhoidectomy and had routine histopathological examination.

Characteristics	Number	Percentage
Gender		
Male	161	48.1%
Female	174	51.9%
Age (years)		
Median	47	
Range	39–59	
Risk group		
Low‐risk patients	309	92.2%
High‐risk patients	26	7.8%
Risk factors		
HIV‐positive	2	0.6%
HPV‐associated infection (CIN, VIN, condyloma)	13	3.9%
Immunosuppression (steroids, biologics, chemotherapy)	11	3.3%
MSM	2	0.6%

*Note*: The percentage of the entire cohort of 335 patients is presented in parentheses.

Abbreviations: CIN, cervical intraepithelial neoplasia; HIV, human immunodeficiency virus; HPV, human papilloma virus; MSM, men who have sex with men; VIN, vaginal intraepithelial neoplasia.

From the final cohort, a combined overall total of 27 (8.1%) patients had abnormal histopathological findings of aSIL and aSCC. These findings were entirely incidental, as none of these patients had suspicious clinical examination findings during preoperative and intraoperative assessment. These abnormal histopathological findings consisted of 19 low‐grade squamous intraepithelial lesions (LSILs) (5.7%), seven (2.1%) high‐grade squamous intraepithelial lesions (HSILs) and one case (0.3%) of aSCC. The only case of incidentally detected aSCC was found in a low‐risk patient.

The risk profile of all patients with abnormal histopathology was assessed and categorized as low risk and high risk (Table 4). Low‐risk patients accounted for 77.8% of the cases, predominantly presenting with LSILs, whereas high‐risk patients made up 22.2%, with the majority exhibiting HSILs. Comparative analysis revealed that the incidental detection rate of aSIL and aSCC was 6.8% (95% CI 4.49–10.17) among all low‐risk patients, compared to 23.1% (95% CI 11.03–52.05) in all high‐risk patients. Additionally, the incidental detection rate of HSIL or aSCC was 1% (95% CI 0.30–2.80) in the low‐risk group and 19% (95% CI 8.50–37.90) in the high‐risk group.

**TABLE 4:** Subcategorization of patients with incidentally detected abnormal histopathological findings based on their risk status.

Histopathology	Low‐risk patients	High‐risk patients	Total (%)
LSIL	18 (66.7%)	1 (3.7%)	19 (70.4%)
HSIL	2 (7.4%)	5 (18.5%)	7 (25.9%)
aSCC	1 (3.7%)	0 (0.0%)	1 (3.7%)
Total	21 (77.8%)	6 (22.2%)	27 (100%)

*Note*: Data are presented as *n* (%) of the overall subset (*n* = 27). Low‐risk patients did not have risk factors related to human papilloma virus infection, anal dysplasia or anal malignancy. Patients considered high risk included HIV‐positive patients, patients with previous HPV‐associated infection, immunosuppression and men who have sex with men. The percentage of the cohort with abnormal histology is presented in parentheses.

Abbreviations: aSCC, anal squamous cell carcinoma; HSIL, high‐grade squamous intraepithelial lesion; LSIL, low‐grade squamous intraepithelial lesion.

The prevalence of each risk factor was compared between groups with normal and abnormal histology using Fisher's exact test. Statistically significant associations were found in patients with a history of HPV infection (*P* = 0.0146) and when all four conventional risk factors were combined (*P* = 0.0116). No significant association was observed for the other independent risk factors (Table 5). Multivariate logistic regression analysis demonstrated that no independent risk factor significantly increased the likelihood of detecting aSIL and aSCC after routine histopathology examination was performed following excisional haemorrhoidectomy (Table 6). Only when all conventional risk factors were combined did a large statistically significant association emerge (OR 3.76; 95% CI 1.32–10.68, *P* = 0.013; Table 7).

**TABLE 5:** Comparison of the prevalence of patient characteristics and risk factors between patients with benign histopathological results and patients with incidental anal dysplasia or malignancy.

Demographic and risk factor	Histology result: benign (308)	Histology result: aSIL/aSCC (27)	*P* value
Median age at surgery	47 (39–59)	47 (38–55)	0.7527
Female	157 (50.8%)	17 (63%)	0.3153
Current smoker/ex‐smoker	147 (47.7%)	14 (51.9%)	0.6935
Aboriginal or Torres Strait Islander origin	13 (4.2%)	0 (0%)	0.6105
HIV‐positive	1 (0.3%)	1 (3.7%)	0.1549
Known history of HPV	**9 (2.9%)**	**4 (14.8%)**	**0.0146**
MSM	1 (0.3%)	1 (3.7%)	0.1549
Immunosuppression	9 (2.9%)	2 (7.4%)	0.2193
Combined ‘high‐risk’ risk factors: HIV/HPV/MSM/immunosuppression	**20 (6.5%)**	**6 (22.2%)**	**0.0116**

*Note*: Percentage is presented in parentheses. Statistically significant results (*P* value ≤0.05) are highlighted in bold. Fisher's exact test was used for two‐group comparison.

Abbreviations: aSCC, anal squamous cell carcinoma; aSIL, anal squamous intraepithelial lesion; HIV, human immunodeficiency virus; HPV, human papilloma virus; MSM, men who have sex with men.

**TABLE 6:** Multivariate logistic regression analysis of patient characteristics and HPV status associated with incidental detection of anal dysplasia or malignancy when routine histopathological examination of haemorrhoid tissue is conducted.

Variable	OR	95% CI	*P* value
Known history of HPV	3.337	0.781–14.247	0.104
Age at admission	0.996	0.967–1.025	0.767
Gender	1.782	0.714–4.450	0.216
Current smoker/ex‐smoker	1.365	0.5778–3.227	0.478

*Note*: No statically significant results were observed.

Abbreviation: HPV, human papilloma virus.

**TABLE 7:** Multivariate logistic regression analysis of patient characteristics and combined four risk factors associated with incidental detection of anal dysplasia or malignancy when routine histopathological examination of haemorrhoid tissue is conducted.

Variable	OR	95% CI	*P* value
Combined ‘high‐risk’ risk factors: HIV/HPV/MSM/immunosuppression	**3.761**	**1.324–10.680**	**0.013**
Age at admission	0.100	0.973–1.027	0.980
Gender	1.495	0.643–3.479	0.350
Current smoker/ex‐smoker	1.111	0.491–2.513	0.800

*Note*: Statistically significant results (*P* value ≤0.05) are highlighted in bold. Statistically significant results were only observed after ‘high‐risk’ risk factors were combined.

Abbreviations: HIV, human immunodeficiency virus; HPV, human papilloma virus; MSM, men who have sex with men.

All abnormal histopathology results were submitted for secondary review by an independent senior pathologist; the diagnoses of five patients were downgraded as a result. Incorporating these findings with respect to the entire cohort of 335 patients who had routine histopathological evaluation, a revised total of 24 patients (7.2%) had abnormal histopathological findings. This consisted of 18 cases of LSILs (5.4%), five cases of HSILs (1.5%) and one case (0.3%) of aSCC. A detailed summary of the results from the secondary review is presented in Table S1 and the adjusted breakdown of patients with abnormal results according to risk status is presented in Table S2.

### Survey results

Our secondary aims were to perform a city‐wide survey of surgeons to ascertain their current local practices and their estimated prevalence of incidental sinister pathology in haemorrhoid specimens. Face to face interviews were conducted with a participation rate of 100%.

Of 14 surgeons (two board‐certified colorectal surgeons and 12 board‐certified general surgeons), the majority (64.3%) would request routine histopathological evaluation following haemorrhoidectomy, and four (all general surgeons, 28.6%) stated that they do not. One general surgeon declined to comment as their regular clinical practice does not include haemorrhoidectomy. Additionally, 64.3% of surgeons believed that the incidental detection rate of aSIL and aSCC was 1% or less.

Surgeons who routinely requested histopathology mainly cited fear of litigation if neoplasia was missed. Other reasons included previous detection of aSIL and aSCC or influence by senior colleagues and mentors. Surgeons who do not routinely request histopathology shared a common perspective that abnormal pathology should only correlate with abnormal examination findings, and thus they would only request histopathology in the presence of suspicious examination findings. One surgeon was unsure about the clinical relevance of aSIL and did not think that detecting this was useful.

### Cost

The total cost of conducting routine histopathological evaluation per patient was $96.80 AUD ($59.20 EUR, $65.30 USD). The cost included laboratory processing and pathologist reporting costs.

## DISCUSSION AND CONCLUSION

This is the first study to determine the incidental detection rate of aSIL and aSCC in low‐ and high‐risk patients who underwent excisional haemorrhoidectomy. We have demonstrated that the overall prevalence of incidental aSIL and aSCC was 8.1%.

Previous studies investigating the prevalence of abnormal pathology in haemorrhoidectomy specimens have reported rates of 0.3%–3.2% [7, 8, 9, 10] and our higher detection rate of sinister lesions may be reflective of the socioeconomic characteristics of our catchment population. Census data from the Australian Bureau of Statistics have consistently reported lower median weekly household incomes and lower socioeconomic index scores for the people of Townsville compared to most regions in Australia [11]. In this study, 92.2% of patients were classified as low risk and did not have conventional risk factors for anal dysplasia and malignancy. Consistent with this finding, multivariate logistic regression analysis demonstrated that no conventional risk factors independently increased the likelihood of detecting aSIL and aSCC significantly. It is therefore possible that other unrecognized risk factors could be contributing to the risk of developing anal dysplasia. Plausible confounding variables which may have higher rates in people with lower socioeconomic index scores include higher rates of tobacco use, early sexual activity, unprotected sex, co‐infection with other sexually transmitted diseases, poorer hygiene and lower HPV vaccination rates. This suggests that there may be other risk factors within the conventional low‐risk cohort that may account for our high detection rate in low‐risk patients.

The findings of this study are consistent with the popular opinion that patients undergoing elective haemorrhoidectomy are conventionally low‐risk patients, and most patients did not have any conventional risk factors. Haemorrhoidectomy is one of the most frequently performed operations in surgery; it is therefore important to determine if there are tangible benefits of routine histopathology in this large cohort. Whilst high‐risk patients within our cohort were more likely to yield abnormal results or have high‐grade dysplasia detected, a more substantial number of abnormal findings were LSIL occurring in low‐risk patients. Another important consideration is expenditure, including laboratory processing and pathologist reporting costs. The cost of conducting routine histopathological evaluation per patient was $96.80 AUD ($59.20 EUR, $65.30 USD). Therefore, based on the prevalence of abnormal findings and the modest cost of histopathological examination, combined with the potential for early detection of HSIL and aSCC, we cannot recommend restricting routine histopathological examination of haemorrhoidal tissue solely to high‐risk patients.

The largest group of patients with aSIL in our study were low‐risk patients with LSIL. There is no study in the literature which has reported the benefit of formal surveillance programmes for this cohort, and there is no consensus recommendation available to guide management. With time, the majority of LSIL is thought to completely regress or it may remain quiescent. A smaller portion of patients with LSIL are at risk of developing a subsequent diagnosis of HSIL and therefore risk future clinical progression to aSCC [3, 12]. The risk of progression from low‐grade to high‐grade disease is unclear and the clinical implications of LSIL detected even in high‐risk patients are not well defined. Current international guidelines do not currently recommend formal regimented surveillance and treatment for LSIL above regular medical history and physical examination [6, 13]. Contrary to the conventional practice of reassurance and discharge for patients with LSIL, for the last decade our colorectal department has recommended regular surveillance and treatment for all patients with anal dysplasia, including LSIL, through high‐resolution anoscopy guided biopsy and ablation at regular intervals. We will be reporting the findings of our surveillance programme, in low‐risk and high‐risk patients with anal dysplasia, in a subsequent publication.

The recently published findings of the ANCHOR trial indicate that active treatment in HIV‐positive patients with HSILs reduces the progression to anal cancer by 57% compared to active monitoring without treatment [14]. Without active treatment, previous studies indicate that the malignant progression rate of HSILs to aSCC varies between 8.6% and 19.6% during a follow‐up period of up to 5 years. High‐risk patients are acknowledged to be more susceptible to malignant transformation [2, 15, 16]. This evidence base underscores the critical importance of early detection and management of HSILs, particularly in high‐risk populations. Identification of HSILs offers an opportunity to initiate high‐resolution anoscopy surveillance and management, to enhance clinical outcomes and to mitigate the risk of anal cancer development in this vulnerable group. Consequently, the debate regarding the significance of HSIL detection has been substantially resolved.

The city‐wide survey of surgeons revealed a diverse range of practices. Surgeons who routinely requested histopathology mainly cited fear of litigation if neoplasia was missed. We found that a considerable proportion of surgeons do not request routine histopathological evaluation and shared a common view that abnormal pathology should only correlate with abnormal examination findings. Our study challenges this commonly held belief and emphasizes the need for evidence‐based recommendations to drive decisions regarding routine histopathological evaluation, rather than reliance on defensive medical practice or fears of legal repercussions.

The limitations of this study arise from the retrospective and single‐centre study design which relies on the accuracy and detail of electronic medical records. The estimated total prevalence of aSIL within our study is higher than reports from studies in other parts of the world and, as we alluded to above, our patients residing without our institution's catchment area have relatively lower socioeconomic status. We identified 7.8% of patients as high risk, but this figure may be underestimated due to incomplete documentation and ascertainment of HIV status, sexual behaviour and gender orientation (unlike HPV history, which was reliably confirmed through cross‐referencing previous histopathological reports).

Based on our secondary review of histopathology for all 27 abnormal findings, 18.5% of cases exhibited interobserver variability, where secondary review suggested revision of the initial diagnosis. Notably, all variations presented in Table S1 resulted in a downgrade of the histological diagnosis; most variations involved differentiation of LSILs from reactive or inflammatory changes. Such discrepancies in final histopathological interpretation are well documented in the current literature and do not directly reflect the quality of histopathological reporting at our institution, as all assessments were conducted by board‐certified pathologists. Over the 9‐year study period, 10 different board‐certified pathologists generated our initial histopathology reports. For the secondary review, an independent board‐certified pathologist with extensive experience in colorectal pathology was commissioned to ensure a high level of consistency in reassessment. Prior studies have similarly demonstrated significant levels of interobserver variability in aSIL histological interpretation, evenly spread among panels of highly experienced and subspecialized pathologists [17, 18]. This inherent subjectivity could be contributing to ongoing uncertainties regarding the natural history of aSIL and variations in clinical outcomes following different management strategies. Our current inability to accurately define the natural progression of aSILs may largely be due to inconsistent and varying interpretations of anal dysplasia by pathologists [17]. Enhancing diagnostic accuracy may require consensus interpretation by at least two pathologists and more consistent use of adjunctive p16 immunostaining, which remains under‐utilized at our institution. While neither approach serves as a definitive gold standard, they provide a more standardized framework for reducing diagnostic uncertainty. Despite the revised diagnoses from our secondary pathology review, a considerable portion of patients (7.2%) retained a diagnosis of aSIL or aSCC after routine histopathology. Of these, the majority (66.7%) were still low‐risk patients with LSILs (Table S2). The clinical implication of this persistent rate of abnormal findings, even after review, is that selective histopathology assessment post haemorrhoidectomy may be inadequate. While the natural history of aSIL requires further investigation, regular clinical follow‐up is prudent for these patients to enable early detection or progression to high‐grade dysplasia or aSCC.

In conclusion, routine histopathological analysis of haemorrhoidectomy specimens identified non‐negligible rates of anal dysplasia and malignancy. Given the considerable portion of LSILs incidentally detected in low‐risk patients, along with the relatively greater occurrence of high‐grade dysplasia detected in high‐risk patients, we therefore cannot recommend limiting routine histopathological examination of haemorrhoid tissue solely to high‐risk patients. Further research is required to understand the evolution of low‐ and high‐grade aSIL, in low‐ and high‐risk patients. Future allocation of resources toward detection and surveillance can only be justified if we have a clear understanding of how these lesions evolve or regress.

## AUTHOR CONTRIBUTIONS

**Panuwat Pornkul:** Writing – original draft; funding acquisition; investigation; conceptualization; methodology; validation; visualization; writing – review and editing; project administration; formal analysis; data curation; resources. **Guy Lampe:** Investigation; data curation; formal analysis; supervision; validation. **Renae Bertucci:** Data curation; investigation; writing – review and editing; project administration; methodology. **Shehan Wickramasinghe:** Investigation; writing – review and editing; data curation; resources. **Kerenaftali Klein:** Software; investigation; data curation; formal analysis; writing – review and editing. **Chrispen Mushaya:** Conceptualization; supervision; writing – review and editing. **Shinichiro Sakata:** Conceptualization; methodology; supervision; funding acquisition; writing – review and editing; writing – original draft; project administration; resources; investigation; formal analysis; visualization.

## FUNDING INFORMATION

This research was conducted without any external funding support. No industry sponsorship or additional financial resources were received for the study or the preparation of this manuscript. PP is a Master of Philosophy student, supported by the Australian Government's Research Training Programme for tuition fees.

## CONFLICT OF INTEREST STATEMENT

This research did not receive any specific grants from funding agencies in public, commercial or not‐for‐profit sectors. There are no conflicts of interest to disclose. All authors provided intellectual contribution and have adhered to ICMJE guidelines on authorship.

## ETHICS STATEMENT

Ethical approval was obtained from the AQUIRE panel (Audit, Quality and Innovation Review) of the Townsville University Hospital. Informed patient consent was obtained following the ethical standards outlined by AQUIRE.

## Supporting information


Data S1.

## Data Availability

The data that support the findings of this study are available on request from the corresponding author. The data are not publicly available due to privacy or ethical restrictions.
