# Supplementary material for: The value of routine histopathological examination after haemorrhoidectomy in patients at low and high risk of anal squamous intraepithelial lesions and cancer
Source: Colorectal Dis. 2025 Mar 17;27(3):e70056. doi: 10.1111/codi.70056 (PMC11911905; doi:10.1111/codi.70056)
Supplement: Supplementary file 1 — Data S1. [file CODI-27-0-s001.docx]

| **SUPPLEMENTARY TABLE 1** Comparison of initial reported histopathology and secondary independent histopathological review of haemorrhoidectomy specimens | | | | |
| --- | --- | --- | --- | --- |
| Patient Number | Conventional Risk Factor | P16 test | Reported Histopathology | Review of Histopathology |
| 1 | Nil | Positive | AIN grades 2-3 with early invasive squamous cell carcinoma | AIN 3 with early invasive squamous cell carcinoma |
| 2 | Immunosuppression and previous HPV | N/A | AIN grades II to III | AIN grades II to III |
| 3 | Nil | N/A | Haemorrhoids and AIN II | AIN I and II |
| 4 | HIV positive and MSM | N/A | AIN I | AIN I |
| **5** | **Nil** | **N/A** | **AIN I** | **Favour reactive or inflammatory change, no evidence of ASIL** |
| 6 | Nil | N/A | AIN I | AIN I |
| 7 | Nil | N/A | AIN I | AIN I |
| **8** | **Nil** | **Negative** | **AIN I** | **Favour reactive of inflammatory change, no definite ASIL** |
| 9 | Nil | N/A | AIN I | Focal AIN I |
| 10 | Nil | N/A | AIN I | AIN I |
| 11 | Nil | N/A | AIN I | Focal AIN I |
| 12 | Nil | N/A | AIN I | AIN I |
| 13 | Nil | N/A | AIN I | AIN I |
| 14 | Nil | N/A | AIN I | Focal AIN I |
| 15 | Previous HPV | N/A | AIN III | Focal AIN II to AIN III |
| **16** | **Nil** | **N/A** | **AIN I** | **Severe cautery artefact, no definite ASIL** |
| 17 | Nil | N/A | AIN I | AIN I |
| 18 | Previous HPV | Positive | AIN II to III | AIN II to III |
| **19** | **Immunosuppression** | **Negative** | **Specimen 2 probable AIN II** | **Favour AIN I and inflammatory change in specimen 1 and 2** |
| 20 | Nil | N/A | AIN I | AIN I |
| 21 | Nil | N/A | AIN I in specimen 1-3 | Severe cautery artefact in specimen 1-2, AIN I in specimen 3 |
| **22** | **Nil** | **Negative** | **AIN II** | **AIN I** |
| 23 | Nil | N/A | AIN I | AIN I |
| 24 | Nil | N/A | AIN I | AIN I |
| 25 | Nil | N/A | AIN I | AIN I |
| 26 | Nil | N/A | AIN I in specimen 1-3 | AIN I specimen 1 and 3. No obvious AIN seen in spec 2 |
| 27 | Previous HPV | Positive | AIN III | AIN III |
| *Note:* Any discrepancies between the initial histopathology report and secondary review are highlight in bold font  *Abbreviations:* AIN, anal intraepithelial neoplasia; HIV, human immunodeficiency virus; HPV, human papilloma virus | | | | |

| **SUPPLEMENTARY TABLE 2.** Revised subcategorization of patients with incidentally detected abnormal histopathological findings based on their risk status, following secondary review of histopathology. Data is presented as n (%) of the overall subset (n=24) | | | |
| --- | --- | --- | --- |
| Histopathology | Low-risk patients | High-risk patients | Total % |
| LSIL | 16 (66.7%) | 2 (8.3%) | 18 (75%) |
| HSIL | 1 (4.2%) | 4 (16.7%) | 5 (20.8%) |
| aSCC | 1 (4.2%) | 0 (0.0%) | 1 (4.2%) |
| Total | 18 (75%) | 6 (25%) | 24 (100%) |
| Low-risk patients did not have risk factors related to human papilloma virus infection, anal dysplasia or anal malignancy. Patients considered high-risk included HIV-positive patients, patients with previous HPV-associated infection, immunosuppression and men who have sex with men.  *Note:* Percentage of the cohort with abvnormal histology is presented in brackets  *Abbreviations:* LSIL, low-grade squamous intraepithelial lesion; HSIL, high-grade squamous intraepithelial lesion; aSCC, anal squamous cell carcinoma. | | | |
